# Umbilical artery cord blood glucose predicted hypoglycemia in gestational diabetes mellitus and other at-risk newborns

**DOI:** 10.1186/s12902-023-01532-x

**Published:** 2023-12-21

**Authors:** Yuan Wang, Huahua Liu, Leilei Zhang, Xin Wang, Mingbo Wang, Zhifang Chen, Feng Zhang

**Affiliations:** 1 Medical College of Nantong University, 19 QiXiu Road, NanAtong City, Jiangsu Province China; 2 Affiliated Maternal and Child Health Care Hospital of Nantong University, Nantong City, Jiangsu Province China

**Keywords:** Umbilical cord blood, Glucose, Hypoglycemia, Newborn, Gestational diabetes mellitus

## Abstract

**Background:**

To explore the value of umbilical artery cord blood glucose (UACBG) in predicting hypoglycemia in gestational diabetes mellitus (GDM) and other at-risk newborns, and to provide a cut-off UACBG value for predicting hypoglycemia occurrence.

**Methods:**

In this prospective study, we enrolled at-risk infants delivered vaginally, including neonates born to mothers with GDM, premature, macrosomic, and low birth weight. We separated the infants into GDM group and other at-risk group. All subjects underwent UACBG measurement during delivery. Neonatal peripheral blood glucose measurement was performed at 0.5 and 2 h after birth. The predictive performance of UACBG for neonatal hypoglycemia was assessed using receiver operating characteristic curve (ROC), area under curve (AUC), sensitivity, specificity, negative predictive value (NPV) and positive predictive value (PPV).

**Results:**

916 newborns were included, with 538 in GDM group and 378 in other at-risk group. 85 neonates were diagnosed hypoglycemia within 2 h after birth, including 36 belonging to GDM group and 49 to other at-risk group. For hypoglycemia prediction within 2 h, the best cut-off of UACBG was 4.150 mmol/L, yielding an AUC of 0.688 (95% CI 0.625–0.751) and a NPV of 0.933. In detail, the AUC was 0.680 in GDM group (95% CI 0.589–0.771), with the optimal cut-off of 4.150 mmol/L and a NPV of 0.950. In other at-risk group, the AUC was 0.678(95% CI 0.586–0.771), the best threshold was 3.950 mmol/L and the NPV was 0.908. No significant differences were observed between GDM group and other at-risk group in AUC at 0.5 h, 2 h and within 2 h.

**Conclusions:**

UACBG has a high NPV for predicting neonatal hypoglycemia within 2 h after birth. It was implied that individuals with cord blood glucose levels above the threshold were at lower risk for hypoglycemia. UACBG monitoring provides evidence for subsequent classified management of hypoglycemia.

## Background

Hypoglycemia is one common condition among the neonatal population. The fetus relies on the maternal metabolism and placental circulation to provide the necessary ketones, glucose, free fatty and amino acids to satisfy its energy needs. Placenta supplies glucose to fetal circulation directly. When the umbilical cord is clamped abruptly at birth, continuous source of glucose was interruptted, causing a rapid decrease in newborns’ blood glucose concentrations during the first hours of life [1]. However, some neonates go through an enduring and serious hypoglycemia. Constant hypoglycemia is likely to lead to brain cell death and even irreversible cranial nerve injury in newborns [2, 3], having an adverse effect on the intellectual development of newborns. Hypoglycemia affected about 15% of newborns [4], especially those born premature, macrosomic, low birth weight or to mothers with gestational diabetes mellitus (GDM) [5].

GDM is the most prevalent disorder during pregnancies, influencing up to 15–25% of women worldwide [6]. The rate of neonatal hypoglycemia in pregnancies with GDM is 8–30% [7]. Fetal hyperinsulinemia caused by the hyperglycemia in utero increases the incidence of neonatal hypoglycemia in gestations with GDM [8, 9].

Other at-risk newborns, including premature, macrosomic and low birth weight newborns, are also likely to be diagnosed with neonatal hypoglycemia, of which the incidence was approximately 50% [5]. Lower storage of glycogen, increased free insulin, absence of substrate source for gluconeogenesis, and increased insulin sensitivity in these at-risk infants are related to neonatal hypoglycemia [10–16].

Maintaining blood glucose dynamic stability is an important step during the fetal to neonatal transition. Clinically, neonatal hypoglycemia usually shows no obvious symptoms, and the diagnosis mainly depends on neonatal blood glucose monitoring. This raises the question of whether it is feasible to diagnose neonates with the highest risk of hypoglycemia more accurately. It has been suggested by Daria Turner et al. that the screening of neonatal hypoglycemia should be performed within one hour after birth ideally [17]. Additionally, the infections and pain resulting from the invasive nature of glucose monitoring have encouraged researchers to investigate non-invasive approaches to predict blood glucose levels. It has been reported that umbilical cord blood glucose level was associated with neonatal blood glucose [18, 19]. A recent study also revealed that immediate cord blood sampling for blood glucose evaluation among at-risk newborns would be an alternative method for early diagnosis of perinatal asphyxia and hypoglycemia [20]. However, the predictive value of umbilical artery cord blood glucose (UACBG) on neonatal hypoglycemiaremains unclear.

The current study explored the value of umbilical artery cord blood glucose (UACBG) in predicting hypoglycemia in gestational diabetes mellitus (GDM) and other at-risk newborns, and to provide a cut-off UACBG value for predicting hypoglycemia occurrence.

## Methods

### Subjects

This prospective study was conducted between January 2021 and February 2022 on at-risk newborns delivered vaginally at Affiliated Maternal and Child Health Care Hospital of Nantong University. This research was approved by the Hospital Ethics Committee (Ethics No: Y 2,020,039). Inclusion criteria were infants delivered vaginally in singleton gestations, who born premature (< 37 week), macrosomic (> 4000 g), low birth weight (< 2500 g) or delivered by mothers with GDM. Fetal malformation and stillbirth were excluded.

### Grouping

GDM group: All newborns delivered by mothers with GDM were included, regardless of whether they were born premature (< 37 week), macrosomic (> 4000 g), or low birth weight (< 2500 g). Due to the low incidence of pre-pregnancy diabetes mellitus, the risk of neonatal hypoglycemia in pregnancies with pre-pregnancy diabetes mellitus differs greatly from that of GDM. In order to maintain consistency of the research, we excluded infants delievered by women with pre-pregnancy diabetes mellitus. According to The International Association of Diabetes in Pregnancy Study Group (IADPSG) [21], with a 75 g oral glucose tolerance test (OGTT) performed during 24–28 gestational weeks, GDM is diagnosed if at least one value is abnormal (fasting: ≥ 5.1 mmol/L, 60 min: ≥ 10.0 mmol/L and 120 min: ≥ 8.5 mmol/L).

The other at-risk group: Except newborns delivered by women with GDM, other at-risk infants requiring close monitoring of blood glucose, including premature, macrosomic, and low birth weight were included. At-risk newborns were defined by the American Academy of Pediatrics (AAP) Committee on Fetus and Newborn [22], including those born premature (< 37 week), macrosomic (> 4000 g), or low birth weight (full-term infants born < 2500 g).

### Routine management of blood glucose after vaginal delivery

#### Diet

Mothers consumed nourishing and easily digestible food in the morning on the day of delivery, such as chicken soup, wonton, noodles, cake, bread, or dessert, to restore their energy levels. After entering labor, midwives in the delivery room conducted diet management on parturients. During the intervals of contractions, midwives instructed parturients to properly take sports drinks or liquid food, normally containing carbohydrate of 45–60 mg/mL and energy of 850–1100 kj/mL. The types of food were selected according to the maternal wishes.

#### Maternal management of blood glucose during labor

For women diagnosed with GDM, the blood glucose level during labor was aimed to maintain at 3.9 − 6.1 mmol/L (70 − 110 mg/dL). Blood glucose was measured every 2 h during labor. If the blood glucose level was higher than 5.6 mmol/L (100 mg/ dL), insulin was provided. Blood glucose monitoring during delivery wasn’t performed on parturients without GDM.

#### Management of at-risk newborns after delivery

After the birth of at-risk newborns, delayed umbilical cord clamping and radiation body rewarming were performed. Rooming-in was implemented in the delivery room within 2 h of delivery, with a room temperature of 26℃. The first sucking was done within 30 min, and skin-to-skin contact lasted 30 min. We extracted the heel blood of all at-risk infants at 0.5 and 2 h after delivery for rapid bedside blood glucose monitoring. After performing the 30 min routine measurement of the peripheral blood glucose, if the blood glucose was < 2.6 mmol/L, breast feeding support was provided by midwives to prolong sucking time; if the blood glucose was < 2.3 mmol/L, liquid milk was provided. Intravenous glucose infusion of 10% dextrose water of 200 mg /kg was conducted on newborns with constant hypoglycemia to raise their blood glucose concentration to 2.2–2.8 mmol/L.

### Measurement

#### UACBG measurement

After vaginal delivery, as the umbilical cord pulse ceased (1–3 min after birth), 1 ml blood was extracted in umbilical artery with heparinized blood gas needle before ligation. GEM Premier 4000 automatic blood gas analyzer (manufacturer: Instrumentation Laboratory Co.; Country: America) was used for immediate measurement in delivery room, acquiring the level of cord blood glucose by the electrode method. Laboratory professionals conducted the blood glucose measurement.

#### Neonatal peripheral blood glucose measurement

Given the ethics of monitoring blood glucose with venous blood, blood glucose measurement was performed with rapid blood glucose meter. Studies have verified that there is a good correlation between the actual blood glucose concentration and the results of the dipstick test, enabling the discrimination of hypoglycemia. Midwives conducted peripheral blood glucose tests. At 0.5 and 2 h after birth, a needle was used to puncture the medial heel of the newborn, and the first drop of blood was discarded. Then one drop of plantar peripheral blood was collected from all newborns. Blood glucose measurement was performed quickly using Roche Accu-Chek micro blood glucose meter (manufacturer: Instrumentation Laboratory Co.; Country: America). When the heel blood was measured < 2.6 mmol/L with rapid blood glucose meter, blood would be re-extracted from the radial artery. Whole blood glucose concentration was measured with glucose oxidase method to diagnose hypoglycemia.

#### Data collection

The electronic medical record system of the hospital was used to collect basic clinical data such as age, gestational age, parity, BMI (body mass index), length of labor, labor analgesia, newborn birth weight, Apgar score, and the test values of OGTT measured at 24 − 28 weeks, and blood glucose at birth.

### Statistical analyses

The sample size was calculated using Power Analysis and Sample Size (PASS) software to detect an area under the receiver operating characteristic curve (AUROC) of 0.70 given a null hypothesis of an AUROC of 0.5. Considering that the neonatal hypoglycemia rate was approximately 10%, we determined that a minimum of 341 infants (including 31 hypoglycemia neonatal and 310 normal glucose neonatal) were needed in each group to obtain a study power of 80% with an α error of 0.05. Subjects with missing data were not included in the current study.

With data entered by two persons, consistency check was performed. All statistical analyses were conducted with SPSS statistics version 25.0. In terms of continuous variables, the Student’s t-test was applied to compare normal distribution data, which were described as means and standard deviations (SD). Mann-Whitney U test was used to compare non-normal distribution data, which were described with median and interquartile range. In the case of categorical variables, we used Chi-square test. The correlation between UACBG and neonatal blood glucose was analyzed by Pearson linear correlation analysis. Receiver Operating Characteristic curve (ROC) curve and area under curve (AUC) were used to assess the discrimination of cord blood glucose predicting neonatal hypoglycemia.

## Results

### Clinical characteristics of infants

A total of 916 newborns were included in our research, 538 in GDM group and 378 in the other at-risk group. Of the newborns in the other at-risk group, 116 (30.69%) were premature, 218 (57.67%) were macrosomic and 44 (11.64%) were low birth weight. Among the GDM group, maternal ages were older (29.31 ± 3.93 year Vs. 28.43 ± 3.61 year), pre-pregnancy BMI was higher (22.48 ± 4.26 kg/m^2^ Vs. 21.90 ± 3.11 kg/m^2^), the proportion of primipara (70.45% Vs. 62.43%) and female newborns (49.44% Vs. 41.27%) were higher, more patients had a 1 min Apgar score < 7 (1.86% Vs. 0.26%), and the labor process was longer (389.22 ± 180.48 min Vs. 365.20 ± 166.71 min). (Table 1)

**Table 1:** Characteristics of the study population

	GDM group (N = 538)	Other at − risk group (N = 378)	F/t/H	p
**Age (years), mean ± SD**	29.31 ± 3.93	28.43 ± 3.61	-3.527	0.000
**Residence, n (%)**			3.375	0.337
City	344(64.18)	229(60.58)		
Country	131(24.44)	94(24.87)		
Migrant population	24(4.48)	27(7.14)		
Rural population	37(6.90)	28(7.41)		
**BMI (kg/m** ^**2**^ **), mean ± SD**				
Pre-pregnancy	22.48 ± 4.26	21.90 ± 3.11	-2.274	0.023
Antepartum	27.37 ± 3.87	27.51 ± 3.56	0.559	0.577
**Parity and gravidity, n (%)**			9.633	0.008
1	379(70.45)	236(62.43)		
2	138(25.65)	132(34.92)		
≥ 3	21(3.90)	10(2.65)		
**Gestational age (weeks)**, **mean ± SD**	38.55 ± 2.06	38.96 ± 1.60	-3.283	0.001
**Premature delivery, n (%)**			83.700	0.000
< 34 week	17(3.16)	21(5.56)		
35 ~ 36^+ 6^ week	21(3.90)	95(25.13)		
≥ 37 week	500(92.94)	262(69.31)		
**Neonatal gender, n (%)**			5.968	0.015
Male	272(50.56)	222(58.73)		
Female	266(49.44)	156(41.27)		
**Neonatal weight (g), n (%)**			454.858	0.000
< 2500	21(3.91)	78(20.64)		
2501 ~ 3999	488(90.88)	82(21.69)		
≥ 4000	28(5.21)	218(57.67)		
**Apgar score 1 min, n (%)**			4.756	0.032
≤ 7	10(1.86)	1(0.26)		
≥ 8	538(98.14)	377(99.74)		
**Apgar score 5 min, n (%)**			-	1.000
≤ 7	1(0.19)	0(0)		
≥ 8	537(99.81)	378(100)		
**Length of labor (min)**, **mean ± SD**	389.22 ± 180.48	365.20 ± 166.71	-2.047	0.041
**Transferred to NICU**, **n (%)**			3.691	0.055
Yes	30(5.58)	11(2.91)		
No	508(94.42)	367(97.09)		
**Complications**, **n (%)**			1.910	0.591
0	339(63.01)	231(61.27)		
1	182(33.83)	138(36.60)		
≥ 2	17(2.96)	8(2.13)		
**pH of cord blood**, **mean ± SD**	7.23 ± 0.90	7.23 ± 0.83	-0.008	0.994

Two cases are absent of information on residence, Apgar score 1 min was modified by fisher exact test. In addition to GDM, complications included premature rupture of membrane, gestational hypertension, hyperthyroidism, hypothyroidism, oligoamnios, polyhydramnios, ICP, anaemia, and placental abruption. GDM, gestational diabetes mellitus

The value of UACBG in GDM group was higher than that in the other at-risk group(5.06 ± 1.23 mmol/L Vs. 4.68 ± 1.08 mmol/L), and their peripheral blood glucose at 0.5 h (4.12 ± 0.94 mmol/L Vs. 3.81 ± 0.92 mmol/L) and 2 h (3.72 ± 0.71 mmol/L Vs. 3.48 ± 0.65 mmol/L ) postnatal was also higher. There are more cases with neonatal hypoglycemia in the other at-risk group, among which the total number of hypoglycemia within 2 h is 49 (14.33%), while 36 (7.27%) in the GDM newborns. The number of 0.5 h neonatal hypoglycemia is 33 (9.02%) in the other at-risk group and 21 (4.13%) in the GDM group respectively. Regarding 2 h neonatal hypoglycemia, the number is 31 (8.99%) in pregnancies with the other at-risk newborns, and 19 (3.84%) in those with GDM. (Table 2)

**Table 2:** Umbilical cord blood glucose, and 0.5 and 2 h peripheral blood glucose in the two groups

	GDM group (N = 538)	Other at − risk group (N = 378)		F/t	p
Umbilical cord blood glucose (mmol/L)	5.06 ± 1.23	4.68 ± 1.08	-4.567		0.000
0.5 h peripheral blood glucose (mmol/L)	4.12 ± 0.94	3.81 ± 0.92	-4.79		0.000
2 h peripheral blood glucose (mmol/L)	3.72 ± 0.71	3.48 ± 0.65	-5.018		0.000
Difference value of change in blood glucose	0.39 ± 1.01	0.35 ± 0.97	-0.548		0.584
0.5 h hypoglycemia	21(4.13)	33(9.02)	8.749		0.003
2 h hypoglycemia	19(3.84)	31(8.99)	9.621		0.002
Total hypoglycemia within 2 h	36(7.27)	49(14.33)	11.033		0.001

Umbilical cord blood glucose: GDM group (n = 478), Other at-risk group (n = 335); 0.5 h peripheral blood glucose: GDM group (n = 508), Other at-risk group (n = 366); 2 h peripheral blood glucose: GDM group (n = 495), Other at-risk group (n = 345). GDM, gestational diabetes mellitus.

### Correlation between UACBG and postpartum peripheral blood glucose

UACBG was related to 0.5 h peripheral blood glucose (r = 0.574) [GDM group (r = 0.540) and the other at-risk group (r = 0.606)]. When it comes to 2 h peripheral blood glucose, the correlation turned out to be (r = 0.166) [GDM group (r = 0.138) and the other at-risk group (r = 0.154)]. (Table 3)

**Table 3:** The correlation between umbilical cord blood glucose and peripheral blood glucose

	umbilical cord blood glucose in GDM group		umbilical cord blood glucose in other at-risk group		Total umbilical cord blood glucose	
	r	p	r	p	r	p
0.5 h peripheral blood glucose in GDM group	0.540	0.000	-	-	-	
0.5 h peripheral blood glucose in other at-risk group	-	-	0.606	0.000	-	-
Total 0.5 h peripheral blood glucose	-	-	-	-	0.574	0.000
2 h peripheral blood glucose in GDM group	0.138	0.004			-	-
2 h peripheral blood glucose in other at-risk group	-	-	0.154	0.007	-	-
Total 2 h peripheral blood glucose	-	-	-	-	0.166	0.000

GDM, gestational diabetes mellitus.

### UACBG predicting neonatal hypoglycemia

**Fig. 1:**
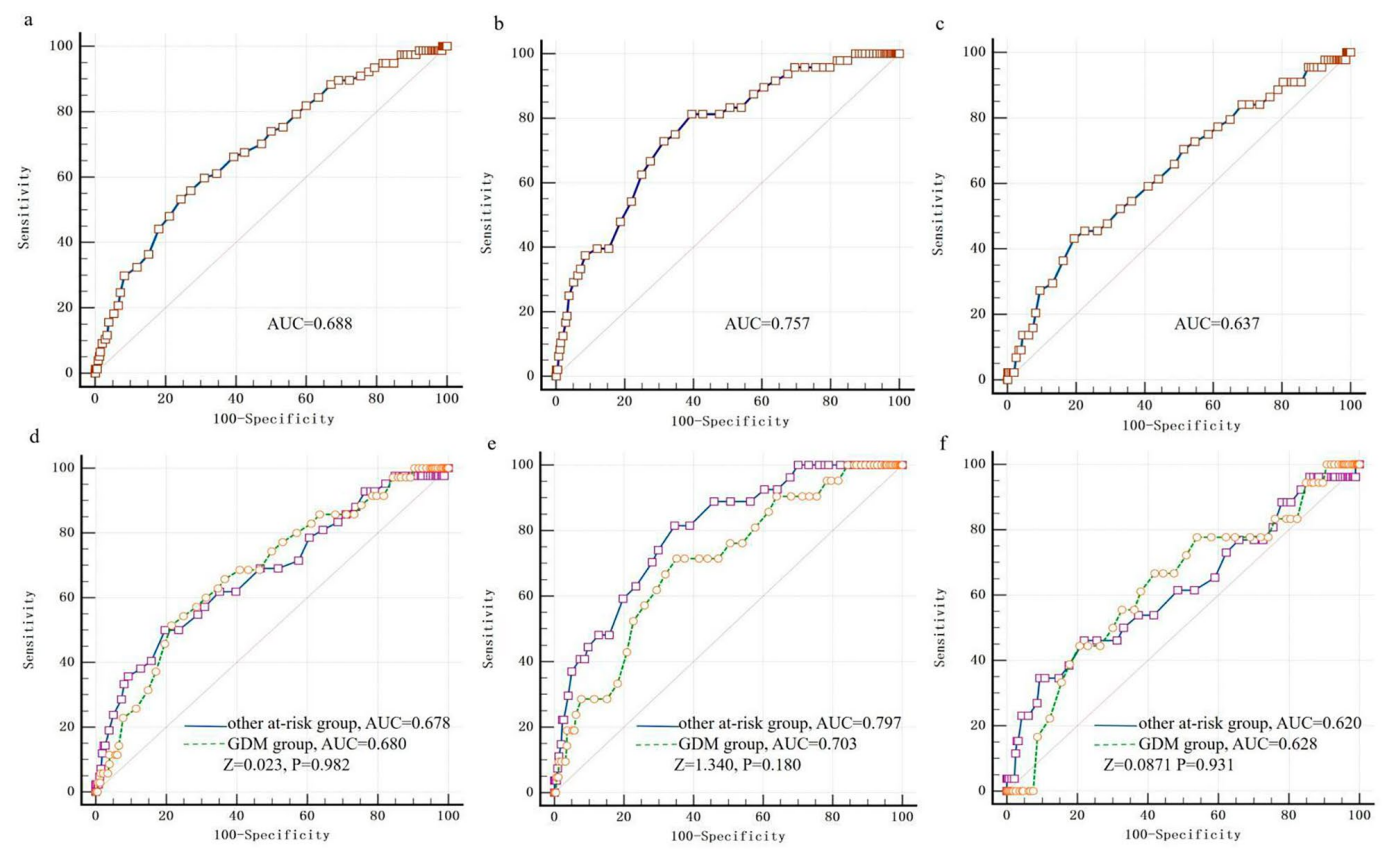
ROC curves for the value of umbilical artery cord blood glucose in predicting hypoglycemia in all newborns; (**a**) within 2 h after birth, (sensitivity: 0.756, specificity: 0.532); (**b**) at 0.5 h after birth, (sensitivity: 0.604, specificity: 0.813); (**c**) at 2 h after birth, (sensitivity: 0.805, specificity: 0.432). Comparison of ROC curves between the GDM group and other at-risk group; (**d**) within 2 h after birth, GDM group (sensitivity: 0.785, specificity: 0.514), Other at-risk group (sensitivity: 0.803, specificity: 0.500); (**e**) at 0.5 h after birth, GDM group (sensitivity: 0.648, specificity: 0.714), Other at-risk group (sensitivity: 0.654, specificity: 0.815); (**f**) at 2 h after birth, GDM group (sensitivity: 0.580, specificity: 0.667), Other at-risk group (sensitivity: 0.906, specificity: 0.346). AUC, area under the curve; GDM, gestational diabetes mellitus

The results of umbilical artery cord blood glucose predicting hypoglycemia are presented in Tables 4 and 5. For total hypoglycemia within 2 h, we found that the best cut-off point was 4.150 mmol/L, yielding a sensitivity of 0.756, a specificity of 0.532, and an AUC of 0.688 (95% CI 0.625–0.751, p = 0.000). The NPV was 0.933 and the PPV was 0.201. At 0.5 h (AUC 0.757; 95% CI 0.690–0.824, p = 0.000), the optimal threshlod was 4.550 mmol/L, with a sensitivity of 0.604, a specificity of 0.813 and a NPV of 0.980. Regarding hypoglycemia at 2 h, we obtained an optimal cut-off of 3.950 mmol/L, yielding a sensitivity of 0.805, a specificity of 0.432, an AUC of 0.637 (95% CI 0.550–0.725, p = 0.002), and a NPV of 0.908. (Fig. 1a-c).

**Table 4:** The results of umbilical blood glucose predicting neonatal hypoglycemia

		Peripheral Venous Blood Glucose						
		Within 2 h			At 2 h		At 0.5 h	
		Hypoglycemia n	Without hypoglycemia n		Hypoglycemia n	Without hypoglycemia n	Hypoglycemia n	Without hypoglycemia n
All newborns	UACBG predicts hypoglycemia	41		163	19	137	39	290
	UACBG predicts no hypoglycemia	36		505	25	567	9	443
GDM group	UACBG predicts hypoglycemia	18		88	12	179	15	153
	UACBG predicts no hypoglycemia	17		321	6	247	6	282
Other at − risk group	UACBG predicts hypoglycemia	21		51	9	26	22	103
	UACBG predicts no hypoglycemia	21		208	17	252	5	195

Hypoglycemia was diagnosed by Peripheral Venous Blood Glucose < 2.6 mmol/L

**Table 5:** Cutoff, sensitivity, specificity, AUC, PPV and NPV for umbilical artery cord blood glucose in predicting hypoglycemia

UACBG predicts hypoglycemia	cut-off, mmol/L	sensitivity	specificity	AUC	P	PPV	NPV
within 2 h in all newborns	4.150	0.756	0.532	0.688 (0.625–0.751)	0.000	0.201	0.933
at 0.5 h after birth in all newborns	4.550	0.604	0.813	0.757 (0.690–0.824)	0.000	0.119	0.980
at 2 h after birth in all newborns	3.950	0.805	0.432	0.637(0.550–0.725)	0.002	0.122	0.958
within 2 h after birth in GDM group	4.150	0.785	0.514	0.680(0.589–0.771)	0.000	0.170	0.950
at 0.5 h after birth in GDM group	4.550	0.648	0.714	0.703(0.594–0.811)	0.002	0.089	0.979
at 2 h after birth in GDM group	4.750	0.580	0.667	0.628(0.496–0.761)	0.066	0.063	0.976
within 2 h after birth in other at-risk group	3.950	0.803	0.500	0.678(0.586–0.771)	0.000	0.292	0.908
at 0.5 h after birth in other at-risk group	4.350	0.654	0.815	0.797(0.716–0.877)	0.000	0.176	0.975
at 2 h after birth in other at-risk group	3.550	0.906	0.346	0.620(0.496–0.744)	0.043	0.257	0.937

GDM, gestational diabetes mellitus; UACBG, umbilical artery cord blood glucose; NPV, negative predictive value; PPV, positive predictive value

Figure 1d-f showed the comparison of ROC curves between the GDM group and other at-risk group. For hypoglycemia prediction within 2 h (Fig. 1d), the AUC was 0.680 (95% CI 0.589–0.771, p = 0.000) in GDM group, with a best cut-off of 4.150 mmol/L and a NPV of 0.950. As to the other at-risk group, AUC was 0.678 (95% CI 0.586–0.771, p = 0.000), the best threshold was 3.950 mmol/L, and the NPV was 0.908. At 0.5 h (Fig. 1e) the NPV was 0.979, the AUC was 0.703 in GDM group (95% CI 0.594–0.811, p = 0.002), and the optimal cut-off point was 4.550 mmol/L, with sensitivity at 0.648 and specificity at 0.714. In the other at-risk group, the sensitivity was 0.654, the specificity was 0.815, and the AUC was 0.797(95% CI 0.716–0.877, p = 0.000), with the best threshold of 4.350 mmol/L and a NPV of 0.975. ROC curves for detection of neonatal hyperglycemia at 2 h were presented in Fig. 1f. The AUC were 0.628 (95% CI 0.496–0.761, p = 0.066) in GDM group and 0.620(95% 0.496–0.744, p = 0.043)in the other at-risk group, with the optimal cut-offs of 4.750 mmol/L (NPV: 0.976) and 3.550 mmol/L (NPV: 0.937). No significant differences were observed between GDM group and the other at-risk group for prediction of neonatal hyperglycemia within 2 h (Z = 0.023, P = 0.982), at 0.5 h (Z = 1.340, P = 0.180) and at 2 h (Z = 0.0871, P = 0.931) after delivery. (Tables 4 and 5)

## Discussion

To our knowledge, this research is the first to explore the value of UACBG in predicting neonatal hypoglycemia in pregnancies with GDM and those with at-risk newborns separately within 2 h after delivery. We found that UACBG was efficient in predicting neonatal hypoglycemia within 2 h after delivery. Routine screening of umbilical glucose has clinical utility for our selected threshold had high negative predictive value, implying high value for excluding neonatal hypoglycemia. No differences were observed between GDM group and the other at-risk group in UACBG prediction.

This study found that the AUC for predicting neonatal hypoglycemia using cord blood was 0.688 within 2 h, 0.757 at 0.5 h and 0.637 at 2 h. Cord blood can be considered as a non-invasive method for predicting neonatal hypoglycemia. Here are some possible theories explaining the pathogenesis of UACBG predicting neonatal hypoglycemia. Placenta glucose transfer [18, 19, 23], DNA methylation from umbilical cord blood and neonatal blood [24], the same source of different cells [25], and Slit-2/Robo1 signaling all might be involved in the pathogenesis of UACBG predicting neonatal hypoglycemia [26]. These evidenc can support our result.

Our result implied that UACBG may be a convenient and non-invasive approach to excluding neonatal hypoglycemia within 2 h after birth, we found that the best cut-off of UACBG was 4.150 mmol/L and the NPV was 0.933 for total hypoglycemia. In GDM group, the best cut-off was 4.150 mmol/L and the NPV was 0.950. In terms of the the other at-risk group, the best threshold was 3.950 mmol/L and the NPV was 0.908. When umbilical cord blood glucose levels are above the cutoff value, a high negative predictive value indicates that it is possible to reduce interventions and glucose tests among this population, helping doctors and healthcare providers make more accurate diagnostic and treatment decisions [27]. However, the relatively low incidence of hypoglycemia may affect the results of negative predictive value. The occurrence of neonatal hypoglycemia in our study is relatively low, the number of which was limited to 85 cases within 2 h after birth. This was due to the management and testing of neonatal blood glucose routine in China. These clinical practice may impact the results of our study, and it should be taken into consideration when promoting our findings clinically.

ROC analyses showed that there were no significant differences in the ability of UACBG predicting neonatal hypoglycemia within 2 h after delivery between GDM group and the other at-risk group, which may be ascribed to diet management on vaginal parturients, early post-delivery breast feeding and optimal maternal glucose management during the antepartum and intrapartum period [28]. However, the umbilical cord blood glucose level is higher in GDM group and the incidence of neonatal hypoglycemia was different between these two groups, which can be explained by the differences in the pathophysiology of hypoglycemia.

The mechanisms of neonatal hypoglycemia in GDM pregnancies are complex. A fetal hyperinsulinemia environment [8, 29, 30], impaired ATP-sensitive potassium channel transition and low cerebro-placental ratio [31–33], and effort made to control maternal blood glucose during labor and delivery [34] all may facilitate hypoglycemia. In terms of the other at-risk group, among premature newborns, hypoglycemia was related to reduced glycogen storage [15, 16]. In macrosomic infants, neonatal hypoglycemia may be ascribed to their excessive growth, improper response to hypoglycemia antenatally, and abnormal distribution of neonatal fat mass and weight. [14, 35]. Infants born with low birth weight have low mobilized energy stores, absence of substrate source for gluconeogenesis, inappropriate secretion of insulin, increased insulin sensitivity, and decreased counter-regulatory hormones [10, 11, 13].

This study has two clinical implications. Firstly, it is recommended that during delivery, cord blood should be collected and blood glucose levels should be measured, which was a non-invasive method to predict the future risk of neonatal hypoglycemia for high-risk infants, including those with GDM, macrosomic, low birth weight, and preterm birth. Secondly, the results of this study suggest that cord blood glucose has a high NPV for predicting neonatal hypoglycemia. Therefore, stratified management should be implemented for newborns. When cord blood glucose is above the cutoff value, it indicates a lower risk of hypoglycemia in the infant. For individuals with cord blood glucose below the threshold, blood glucose monitoring and management should be implemented, including timely initiation of breastfeeding, mother-infant contact, and regular blood glucose monitoring, to prevent neonatal hypoglycemia. Cord blood testing and stratified management of newborns can reduce the pain and infection risks associated with repeated invasive blood glucose monitoring for low-risk infants. The reduction in clinical nursing workload makes it worthy of clinical promotion.

This research has some limitations. The generalizability of our findings may be limited owing to infants born in a single hospital and lack of follow-up. In addition, we acknowledge that intervention thresholds for hypoglycemia are different among institutions, so the definition of ≤ 2.6 mmol/L may cause a bias in the conclusion. Currently, clinical practices regarding blood glucose monitoring and intervention for GDM, premature, macrosomic and low birth weight infants may lead to a decreased incidence of hypoglycemia. What’s more, the relatively low incidence of hypoglycemia in our study may affect the results of negative predictive value. Thus, our findings should be cautiously interpreted.

Nevertheless, in contrast with other studies on the identification of neonatal hypoglycemia, our research has advantages. First, currently invasive neonatal glucose monitoring could lead to breaking of skin, pain and the potential for infection [36]. We met the call for the development of a measurement non-invasively and painlessly evaluating glucose in neonates. We separated modeling of GDM and other at-risk infants, further determining the application scope of UACBG in predicting neonatal hypoglycemia. Second, considering the effect of cesarean delivery on the odds of neonatal hypoglycemia, only vaginal delivery was included, which also increases the reliabiliy of our findings. Third, given the differences in post-delivery breast feeding, formula milk supplementation, and early skin to skin contact among newborns in different medical institutions, we explored the predictive value of cord blood glucose at 0.5 and 2 h respectively.

## Conclusions

UACBG could help predict neonatal hypoglycemia within 2 h after delivery. Cord blood glucose has a high NPV for predicting neonatal hypoglycemia, implying that individuals with cord blood glucose levels above the threshold were at lower risk for hypoglycemia. It is possible to reduce invasive blood glucose monitoring in clinical practice based on cord blood glucose in the future. No significant differences were observed in the ability of UACBG in predicting neonatal hypoglycemia within 2 h after delivery between GDM group and the other at-risk group. It is recommended to adopt this non-invasive method to assess the risk of neonatal hypoglycemia after birth and implement different management strategies for newborns.

## Data Availability

All data generated or analysed during this study are included in this published article.

## Abbreviations

AAP: American Academy of Pediatrics
AUC: Area under curve
AUROC: Area under the receiver operating characteristic curve
DNAm: DNA methylation
GDM: Gestational diabetes mellitus
IADPSG: International Association of Diabetes in Pregnancy Study Group
OGTT: Oral glucose tolerance test
PASS: Power Analysis and Sample Size
ROC: Receiver operating characteristic curve
SD: Standard deviations
UACBG: Umbilical artery cord blood glucose
PPV: Positive predictive value
NPV: Negative predictive value
